# Contrastive prototype federated learning against noisy labels in fetal standard plane detection

**DOI:** 10.1007/s11548-025-03400-6

**Published:** 2025-05-21

**Authors:** Maria Chiara Fiorentino, Giovanna Migliorelli, Francesca Pia Villani, Emanuele Frontoni, Sara Moccia

**Affiliations:** 1 Department of Information Engineering, Università Politecnica delle Marche, Ancona, Italy; 2 Department of Law, Università degli Studi di Macerata, Macerata, Italy; 3 Department of Political Sciences, Communication and International Relations, Università degli Studi di Macerata, Macerata, Italy; 4 Department of Innovative Technologies in Medicine and Dentistry, Università degli Studi “G. d’Annunzio”, Chieti, Pescara, Italy

**Keywords:** Standard-plane detection, Contrastive learning, Ultrasound imaging, Fetal ultrasound

## Abstract

**Purpose:**

This study aims to improve federated learning (FL) for ultrasound fetal standard plane detection by addressing noisy labels and data size variability across decentralized clients. We propose a federated denoising framework leveraging prototypes from the largest dataset in the federation to refine noisy labels and enhance predictions in all clients while preserving privacy.

**Methods:**

The proposed framework consists of two main steps. First, contrastive learning (SimCLR) is applied to the images of the largest client, generating robust embeddings. These embeddings are used to refine noisy labels in the same client by leveraging the latent space structure using a threshold-based k-nearest neighbors re-labeling strategy. As a second step, image prototypes, computed from the embeddings with noise-free labels, along with SimCLR trained backbone, are shared with the smallest client to guide the FL process effectively, without requiring the use of labels from the smallest client. To address possible image distribution shifts, an ensemble strategy is introduced, which uses a majority voting scheme to optimize label refinement in the smallest dataset while minimizing image discard.

**Results:**

Our framework showed improved performance compared to traditional FL approaches in standard plane detection, achieving the highest mean F1-score across planes.

**Conclusions:**

The proposed strategy effectively improves fetal standard plane detection by leveraging high-quality prototypes, enabling robust performance even with noisy and heterogeneous data size across clients, while preserving privacy.

## Introduction

Fetal standard planes, such as the abdominal, brain, and cardiac views, are key in prenatal ultrasound (US) for measuring fetal biometric parameters and identifying abnormalities like growth restrictions and congenital anomalies [1, 2]. Traditionally, locating these planes has relied heavily on the skill of experienced sonographers. However, the subjective nature of manual interpretation can affect the precise identification of these planes, driving the need for deep learning (DL) methods to streamline the automatic identification of these planes and improve the accuracy of biometric measurements [3].

While DL models have shown considerable promise in automating the detection of standard planes, their implementation in clinical practice remains limited. This can be explained by the limited representativeness of training datasets, which often fail to capture the full variability encountered in real-world clinical settings [4]. Most datasets used for training come from single-center studies or involve subjects from specific demographic groups [1, 5, 6], limiting DL model ability to generalize to diverse populations. This can lead to biased predictions and potential disparities in clinical outcomes [7]. To address this limitation, there has been growing interest in multicenter studies, such as those by [8, 9], and [7], which, however, may raise privacy concerns due to the integration of data from multiple centers. Federated learning (FL) has emerged as a promising solution to mitigate this issue, enabling collaborative model training across multiple institutions without sharing local images [10]. This decentralized approach preserves patient privacy and facilitates the inclusion of data from multiple hospitals, potentially enhancing models’ robustness and generalizability. At the same time, the involvement of multiple hospitals in an FL setting introduces new challenges, such as different dataset sizes among clients [11], potentially leading to imbalanced contributions to the global model, with biased performance favoring clients with larger datasets, and variation in data labeling quality [12]. Today, there is an established literature relevant to centralized learning with noisy labels [13] but centralized noise filtering algorithms cannot always be exploited in multi-centric studies due to privacy concerns. In other fields of medical image analysis, researchers have started to explore techniques to handle noisy labels in FL [14] but the problem of different dataset sizes among clients is not considered.

In this work, we address the problem of federated fetal standard plane detection in the presence of noisy labels from a large (5187 images) [15] and a small (450 images) dataset [8] from two different continents (Europe and Africa, respectively).

Our strategy begins by leveraging the largest and, therefore, presumably the most representative client in the federation to extract robust embeddings using contrastive learning. Our idea is that these embeddings should capture the most significant geometrical features inherent to each standard plane (i.e., brain, femur, abdomen, thorax), as preliminarily shown in [1], and can be used to refine noisy image labels of the same client. Prototypes computed from the noise-free embeddings, along with the backbone trained via contrastive learning, are shared with the smallest client to label it robustly and guide the learning process in the federation.

We show that we can mitigate the impact of noisy labels in clients with different data size, improving the overall performance of standard plane detection across the federation.

**Fig. 1:**
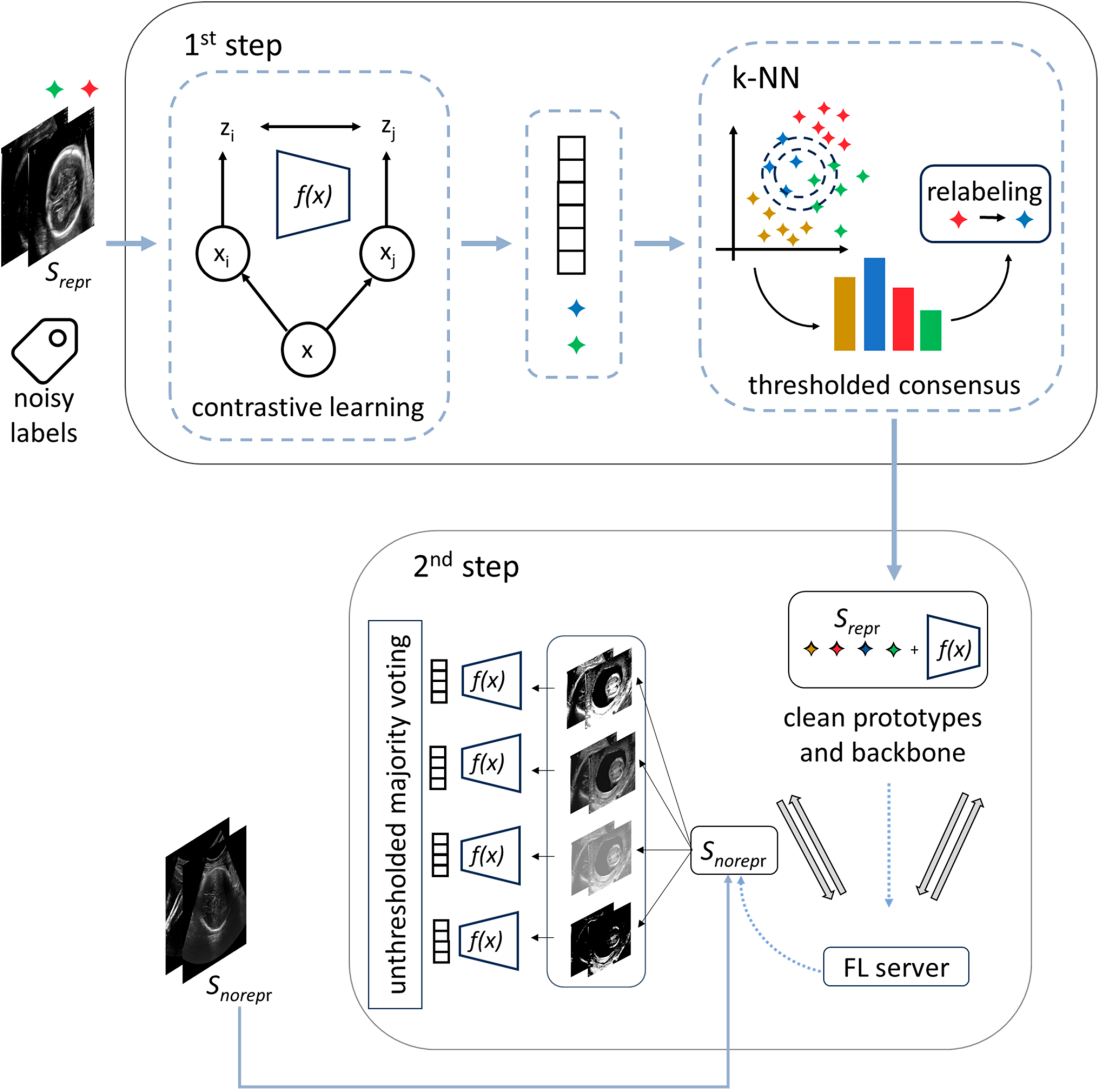
Overview of the proposed federated learning framework. In the first step, contrastive learning (SimCLR) is used on the largest dataset in the federation ($S_{\text {repr}}$) to produce embeddings. A k-NN-based thresholded consensus is used for re-labeling $S_{\text {repr}}$ samples in the embedding space, resulting in a noisy label-free $S_{\text {repr}}$. In the second step, prototypes computed from clean embeddings, along with the SimCLR backbone pretrained self-supervisely on $S_{\text {repr}}$ (*f*(*x*)), are shared through the federated learning server with the smallest dataset ($S_{\text {norepr}}$). Multiple augmented views of $S_{\text {norepr}}$ are generated, their embeddings are extracted, and unthresholded majority voting is used to assign labels to $S_{\text {norepr}}$ based on the clean prototypes

## Method

Figure 1 shows an overview of the proposed framework. The first step involves applying contrastive pre-training using SimCLR [16] on the largest, most representative client ($S_{\text {repr}}$) to learn robust noise-free embeddings. Following SimCLR, given an input image ($x$), we generate two augmented views of it, denoted by $x_i$ and $x_j$, by applying two random transformations ($R_i(x)$ and $R_j(x)$). Both the augmented images are fed into a backbone encoder network ($f(\cdot )$), which maps them into a latent space in the form of two embeddings ($z_i = f(x_i)$ and $z_j = f(x_j)$). $f(\cdot )$ is trained to maximize the agreement between these two embeddings in the latent space, encouraging invariant representations learning of the input, while maximizing the distance from negative pairs to prevent complete collapse. This is achieved by minimizing:

1 $$\begin{aligned} \mathcal {L}_{\text {SimCLR}} = -\log \frac{\exp (\text {sim}(z_i, z_j)/\tau )}{\sum _{r=1}^{2N} \mathbb {1}_{[r \ne i]} \exp (\text {sim}(z_i, z_r)/\tau )} \end{aligned}$$

where $\text {sim}(z_i, z_j)$ represents the cosine similarity between the embeddings $z_i$ and $z_j$, $\tau$ is the contrastive temperature parameter, $\mathbb {1}$ is the indicator function, which is equal to 1 if $y_j = c$ and 0 otherwise, and $N$ is the batch size. By leveraging the structure of the latent space resulting from SimCLR, we use k-nearest neighbors (k-NN) to refine noisy labels in $S_{\text {repr}}$. For each image embedding, we identify its *k*-nearest neighbors based on the Euclidean distance. A threshold (*th*) is further set so that a sample is kept if at least the *th*% of its k-nearest neighbors shares the same label (there is a consensus), otherwise the sample is discarded. We choose the value of *th* as a trade-off between minimizing the number of samples with noisy labels and maximizing the number of samples to be kept.

In the second step, we focus on the client with the smallest sample size ($S_{\text {norepr}}$). Instead of performing re-labeling as done for $S_{\text {repr}}$, we directly discard $S_{\text {norepr}}$ labels. In fact, $S_{\text {norepr}}$ may struggle to benefit from the self-supervised learning approach applied in the first step due to its limited size [16]. To comply with the privacy-preserving nature of FL, our strategy only shares the pretrained backbone $f(\cdot )$ from the first step and class-specific prototypes computed from $S_{\text {repr}}$, where the prototype ($p_c$) for class $c$ is computed as:

2 $$\begin{aligned} p_c = \frac{1}{\mathcal {D}_c} \sum _{z \in \mathcal {D}_c} z \end{aligned}$$

with $\mathcal {D}_c$ being the set of embeddings labeled as $c$.

These prototypes act as compact, privacy-compliant feature representations, guiding $S_{\text {norepr}}$ to align with the knowledge captured in the initial phase. To ensure robustness to possible variability in image acquisition across centers, we apply several augmentations to each image from $S_{\text {norepr}}$. For an image $x \in S_{\text {norepr}}$, we generate $T$ augmented views ($\{x_1, x_2, \dots , x_T\}$) through operations such as random flips, rotations, and brightness changes. These transformations are denoted as:

3 $$\begin{aligned} \mathcal {A}(x) = \{ R_1(x), R_2(x), \dots , R_T(x) \} \end{aligned}$$

where each $R_i(x)$ represents the transformation applied to the image $x$, with $R_1(x)$ being the identity transformation. Each augmented image is fed to the shared $f(\cdot )$, which extracts the corresponding embedding. This results in a set of embeddings $\{z_1, z_2, \dots , z_T\}$. For each extracted embedding, the closest $S_{\text {repr}}$ prototype within the latent space is identified, and its label is assigned to it. The final sample label ($\hat{y}$) is assigned based on the class that receives the majority of votes among the embeddings for that sample:

4 $$\begin{aligned} \hat{y} = \arg \max _{c} \sum _{z_j \in \mathcal {N}(z_i)} \mathbb {1}[y_j = c] \end{aligned}$$

where $\mathcal {N}(z_i)$ denotes the neighborhood of the embedding $z_i$, consisting of the closest embeddings to $z_i$ in the feature space.

**Table 1:** Dataset information in terms of Country, device, and standard planes

Country	Device	Abdomen	Brain	Femur	Thorax
$$S\_repr$$					
Spain	Voluson E6, Voluson S8, Voluson S10, Aloka	711	1718	1040	1718
$$S\_norepr$$					
Malawi	Mindray DC-N2	25	25	25	25
Egypt	Voluson P8	25	25	25	25
Uganda	ACUSON X600	25	25	25	0
Ghana	EDAN DUS 60	25	25	25	0
Algeria	Voluson S8	25	25	25	25

### Datasets

We here use two multi-centric datasets, which correspond to $S_{\text {repr}}$ and $S_{\text {norepr}}$. Table 1 provides an overview of dataset size, Countries involved, number of fetal US images available for the anatomical planes (i.e., abdomen, brain, femur, and thorax), and acquisition devices used.

**Dataset 1:** This dataset acts as $S_{\text {repr}}$ and is a publicly available dataset collected from two centers in Spain, released by [15]. Several operators with similar experience acquired fetal US images from six different US machines including three Voluson E6, one Voluson S8, one Voluson S10, and one Aloka. The images are acquired using a curved transducer with a frequency range of 3 to 7.5 MHz for abdominal US, and a 2 to 10 MHz vaginal probe for cervical US screening during the second and third trimesters.

**Dataset 2:** This dataset acts as $S_{\text {norepr}}$ and is a publicly available dataset with images collected from 5 African countries (Malawi, Algeria, Uganda, Ghana, and Egypt), released by [8]. Different operators acquired the images using US scanners from various vendors, including GE Medical Systems, Siemens, Edan Instruments, Shenzhen Mindray Bio-Medical Electronics and Aloka. The acquisition was done using a curved transducer with a frequency range of 3 to 7.5 MHz, during the second and third trimester of pregnancy.

### Experimental settings

For SimCLR, ResNet-50 is used as $f(\cdot )$. A projection head with a single nonlinear layer comprising 256 nodes processes embeddings of length 2048 obtained from the final average pooling layer. The batch size *N* is set to 256, and the temperature $\tau$ is set to 0.5 as in [1]. For k-NN classification, the value of *k* is set to 50, while *th* is set to 40.

To set the value of *th*, we take into consideration values from 40 to 70 with steps of 10 and assess, at varying levels of noise (0%, 20%, 50%), the trade-off between the number of preserved samples and the percentage of residual noisy labels. We consider 40 as a minimum value to ensure enough agreement among the 4 classes within the neighborhood.

For the transformations in Eq. (3), we use random horizontal flips, rotations up to 15 degrees, and shifts up to 12 pixels along both the x- and y-axes. Brightness and contrast adjustments are performed within a range 0.7 to 1.3 to simulate diverse lighting conditions. Linear mix-up ($\alpha = 0.5$) is used for regularization. For both datasets, we follow the train-test split proposed in the original papers presenting the datasets. We further split the training set of each dataset, allocating 20% of the samples to a validation set to monitor the learning process and detect potential overfitting during training. Following [17, 18], for simulating label noise, we consider uniformly distributed noise among all classes. Model updates from the local clients are aggregated at the central server using federated averaging (FedAvg). Training is conducted over 5 communication rounds, each with 20 local epochs. The number of rounds is set to 5 based on the minimum validation loss observed when running the FL process up to 100 rounds. While increasing the number of epochs per round can reduce communication overhead, excessive epochs in clients with high inter-client heterogeneity may hinder convergence [19]. For this reason, we set 20 epochs per round, the minimum required to complete a full cycle of the learning rate scheduler. The optimization process uses the stochastic gradient descent (SGD) optimizer with a cosine annealing warm restart scheduler. The scheduler starts with an initial learning rate of 0.05, which is reduced to 0.00001 within 20 epochs (1 round). Warm restart applies at the beginning of the next round. The batch size is set to 16, and optimization is performed using the standard cross-entropy loss function. The entire framework is built in Python 3.8.10, using PyTorch 2.0.0 and Torchvision 0.15.1. Computations are distributed across 4 NVIDIA A100 GPUs, each equipped with 64GB of VRAM, on a system with 512GB of RAM, ensuring efficient and scalable training.

### Ablation study

The performance of our framework was first compared with that of traditionally (i.e., locally) trained models (Local_train) in the presence of noise, as to evaluate the potential gain of training a joint, federated model specifically designed to address noise-related challenges.

As a first experiment (Simple_FL) for FL, we analyzed the performance of FedAvg under varying noise levels (0%, 20%, 50%) in $S_{\text {repr}}$ and $S_{\text {norepr}}$ to assess how noise affects the detection performance independently of the proposed strategy for cleaning image labels in the first step of our framework.

**Fig. 2:**
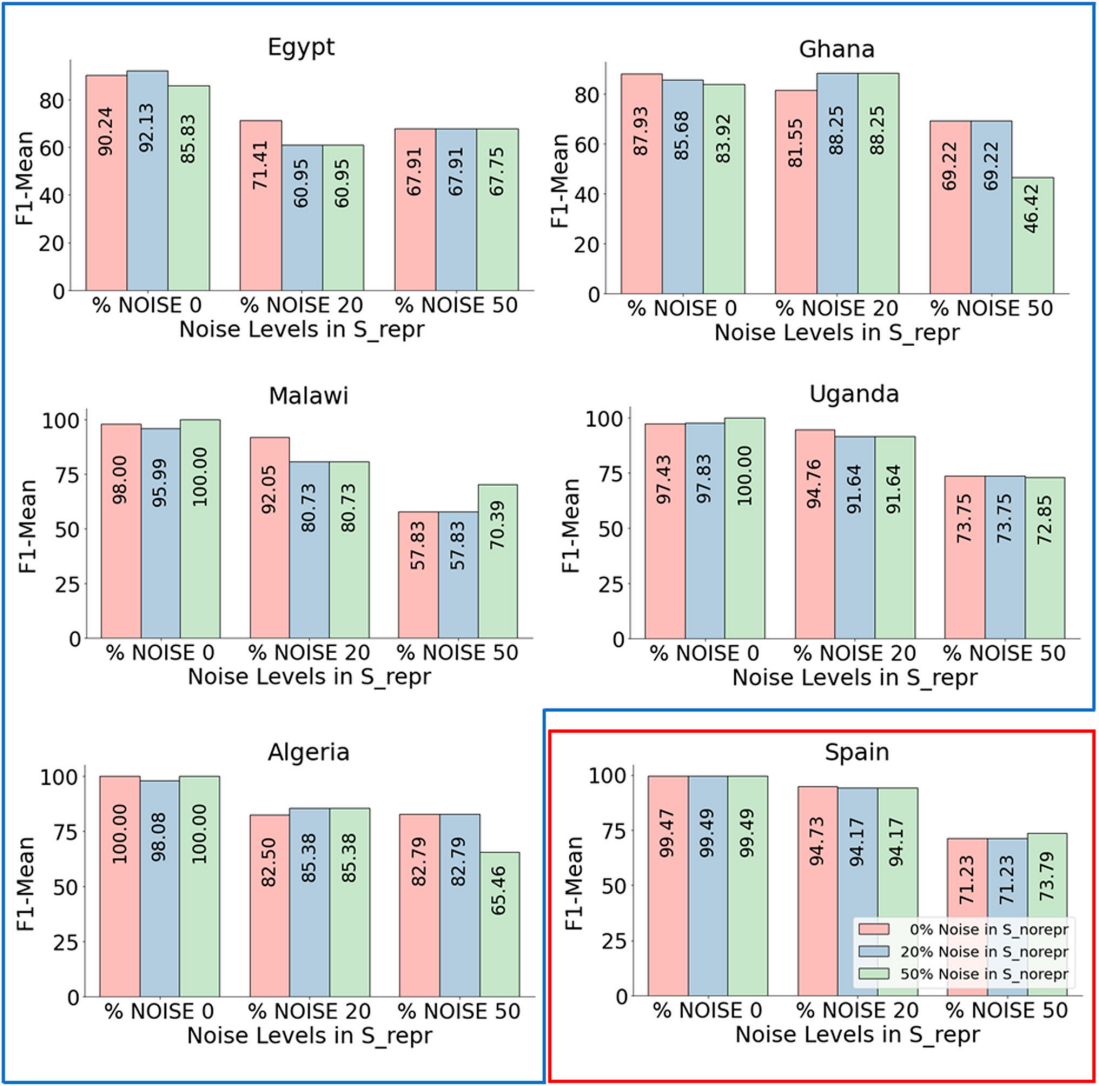
Impact of varying noise levels (0%, 20%, and 50%) in the $S_{\text {repr}}$ dataset (x-axis) on the F1-mean scores across different Countries for Simple_FL. The African countries belonging to the $S_{\text {norepr}}$ dataset (Egypt, Ghana, Uganda, Malawi, Algeria) are highlighted with a blue box, while Spain, the $S_{\text {repr}}$ dataset is highlighted with a red box

To assess the impact of the second step of our framework, we used FedAvg, filtered the noisy labels in $S_{\text {repr}}$ and introducing 0% (Baseline) 20% (Baseline20) and 50% (Baseline50) of label noise in $S_{\text {norepr}}$. Prototype sharing was evaluated in the Proto+Baseline configuration, i.e., we excluded the use of views from the proposed framework to check if this was enough to tackle possible variability among image acquisition protocols across centers.

The combination of prototypes with multiple views outside FL was explored in the Proto+views experiment, where training was performed on $S_{\text {repr}}$ and $S_{\text {norepr}}$ was classified based on $S_{\text {repr}}$ prototypes using augmented views. This experiment aimed to assess whether differences between the two datasets, potentially caused by varying acquisition protocols, could be mitigated through the application of transformations and comparison against prototypes, without requiring $S_{\text {norepr}}$ to participate in the federated learning process.

As a last experiment, we assessed the performance of $f(\cdot )$ trained on $S_{\text {repr}}$ with noise-free labels and tested on $S_{\text {norepr}}$. This experiment (Pretrained weights) assessed the potential benefits of transferring knowledge from a clean, representative dataset on a small one.

## Results and discussion

Results from Local_train are shown in Table 2. With such a simple training strategy, $S_{\text {repr}}$ and, more evidently, $S_{\text {norepr}}$ experienced a drastic drop in performance at high noise levels. This first result emphasizes the need of (i) guiding the training on $S_{\text {norepr}}$ via the larger $S_{\text {repr}}$ dataset and (ii) exploiting noise filtering.

**Table 2:** F1-score averaged over all classes for $S_{\text {repr}}$ and $S_{\text {norepr}}$ for Local_train

%noise	$S_{\text {repr}}$	$S_{\text {norepr}}$				
	Spain	Algeria	Egypt	Ghana	Malawi	Uganda
0	99.40	96.15	96.15	84.56	100.0	100.0
20	93.02	86.38	94.15	86.90	82.90	100.0
50	60.48	36.15	59.45	59.68	60.83	61.00

Moving to Simple_FL, Fig. 2 shows the F1-score obtained for $S_{\text {repr}}$ and $S_{\text {norepr}}$ with varying noise levels for each client. The figure presents the results obtained setting noise levels at 0%, 20%, and 50% in $S_{\text {repr}}$, while independently varying the noise levels in $S_{\text {norepr}}$ across the same range. The results for $S_{\text {norepr}}$ are reported individually for images acquired from Egypt, Ghana, Malawi, Uganda and Algeria, as they may exhibit differing image distributions. The impact that the noise in $S_{\text {norepr}}$ had on $S_{\text {repr}}$ did not appear to be particularly significant, as the client generally maintained performance within the same order of magnitude across varying noise levels. This observation supports the initial intuitive hypothesis of this study: knowledge transfer is predominantly driven by the most representative client. Clients with smaller datasets benefit from the influence of the representative client, as their learning process is positively guided, even in the presence of noise from incorrectly labeled data.

Baseline showed to be robust to noise injected to $S_{\text {repr}}$. We only had a slight decline in performance when 20% of its data was mislabeled. However, performance deteriorated significantly as noise levels increased to 50%. As expected, the noise introduced in $S_{\text {norepr}}$ did not appear to affect the performance on $S_{\text {repr}}$. Overall, the performance of Baseline on $S_{\text {repr}}$ remained superior to that of Local_train (shown in Table 2).

As shown in Table 3, using *th* = 40 allowed the proposed framework to obtain a clean version of $S_{\text {repr}}$, preserving 93% of samples with 20% noise injected and 76% with 50% noise injected. This threshold was selected because the higher tested thresholds (i.e., 50, 60, and 70) drastically reduced the percentage of preserved samples as follows: under 50% noise (36%, 3%, and 0%, respectively) and 20% noise (77%, 64%, and 46%, respectively).

As shown in Table 4, as the filtering is applied in $S_{\text {repr}}$, with Baseline we achieved a mean F1-score of 93.26% under noise-free conditions. However, as noise is introduced in $S_{\text {norepr}}$, the decline in performance remains relatively controlled. With Baseline20, the mean F1-score drops to 92.86%, and even Baseline50, the model maintains a mean score of 91.12%. This highlights the robustness of FedAvg, largely attributed to the presence of a clean, representative client ($S_{\text {repr}}$), which plays a crucial role in mitigating the impact of noise on smaller datasets. The pivotal role of this dataset is further evidenced by pretrained weights experiment, where, even in the absence of labels, the model maintains a relatively high performance, achieving a mean F1-score of 92.35%.

**Table 3:** Effects of label denoising applied to $S_{\text {repr}}$ with varying percentage of noise (% noise) in terms of percentage of preserved samples

%noise	Before denoising	After denoising	
	#samples	#samples	%preserved samples
0	2840	2706	95
20	2840	2646	93
50	2840	2169	76

**Table 4:** F1-score averaged over all classes for the ablation study

Method	% noise	mean-F1 over classes by country						
		Algeria	Egypt	Malawi	Uganda	Ghana	Mean	$\uparrow \downarrow$
Baseline	0	96.13	87.99	84.17	98.00	100.00	93.26	0
Baseline20	20	94.03	90.12	84.17	95.99	100.00	92.86	$-$ 0.40
Baseline50	50	94.03	85.81	81.85	93.89	100.00	91.12	$-$ 2.14
Proto+Baseline	no labels	93.96	81.34	88.43	98.00	98.67	92.08	$-$1.18
Proto+views	no labels	100.00	80.53	90.87	95.99	97.43	92.97	$-$0.29
Proposed framework	no labels	98.10	79.49	94.87	100.00	100.00	94.49	**+1.23**
Pretrained weights	–	98.00	86.15	83.49	98.00	96.10	92.35	$-$0.91

**Fig. 3:**
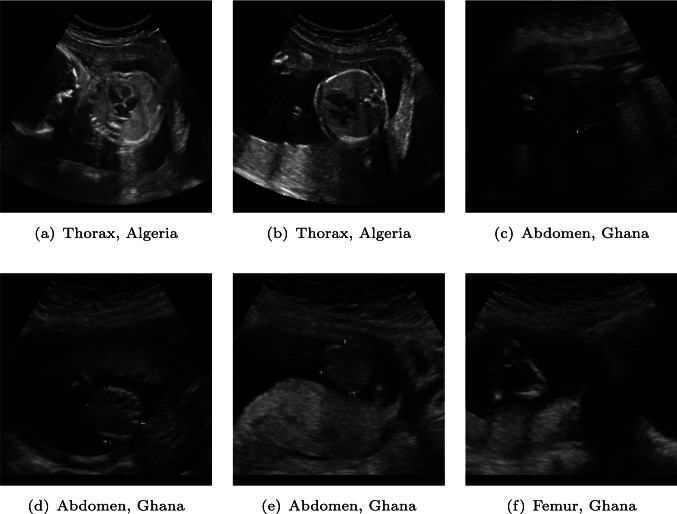
Samples where Baseline fails, while the proposed framework provides correct results. **a** Baseline incorrectly classified the sample as abdomen, overlooking crucial features such as the ventricles; **b** Baseline erroneously predicted the sample as brain; **c** the prediction is femur, likely influenced by the contrasted upper part of the abdomen; **d** the sample is predicted as brain by Baseline, possibly misled by its shape; **e** the sample is predicted as femur as for c; **f** is completely misunderstood as thorax by FedAvg

When Proto+Baseline was tested, we saw that incorporating prototypes into the FL process gave mixed results. The overall mean F1-score reached 92.08%, showing an improvement over Baseline50 but a slight decline compared to noise-free Baseline. This suggests that incorporating prototypes may not be enough to mitigate the effects of noise without additional refinement, as performance can be impacted by potential shifts introduced by different vendor machines. In fact, the Proto+views approach achieved a better mean F1-score of 92.97%. This highlights the potential performance boost that can be achieved by addressing potential distribution shifts by using augmented views.

The most promising results come from the proposed framework, which integrates prototypes, views, and FL. This approach achieved the highest mean F1-score of 94.49% (+1.23% over Baseline). The improvement stemmed from the complementary strengths of the components. The prototypes provided a stable, noise-resistant foundation by leveraging robust embeddings from the large and clean dataset, effectively transferring knowledge to the small client. The ensemble views further enhanced the performance by introducing diverse perspectives for each sample, helping to smooth out inconsistencies or shifts in data distributions. Finally, federated averaging ensured that all clients benefitted from shared global knowledge. Figure 3 shows that our framework enabled a robust extraction of discriminative features, being able to capture key characteristics, such as roundness of brain, the straight line of femur, and chamber shape of the thorax.

As a future improvement, it would be valuable to investigate how the proposed framework performs in scenarios with low inter-class variability, such as in fetal standard plane brain images [20]. Additionally, testing different backbone architectures could further enhance the framework performance while also exploring strategies such as knowledge distillation or edge computing approaches to adapt the proposed framework for environments with limited computational resources. Beyond FedAvg, exploring alternative aggregation methods, also specifically tailored for handling non-IID data such as FedProx [19], SCAFFOLD [21], FedRoD [22], could help optimize the robustness of the FL process. Lastly, in this work, we adopted a symmetric noise model, assuming uniformly distributed noise across all classes. While this approach facilitates the analysis and aligns with prior studies [17, 18], it may not fully capture the complexity of practical noise patterns. Future work will focus on investigating the impact of asymmetric and class-dependent noise distributions on the model performance, to provide a more comprehensive understanding of its generalization capabilities.

## Conclusion

This study showed the effectiveness of leveraging prototypes and ensemble views into FL for fetal standard plane detection, particularly in scenarios with unbalanced client representativeness and varying levels of label noise. Our results revealed that traditional FL methods are moderately robust to noise and their performance declines significantly as noise increases across clients. In contrast, our framework presented substantial improvements in performance, offering a promising solution for fetal standard plane detection, paving the way for more reliable and accurate diagnostic support systems in clinical practice.

## Declarations

### Open Access

All data used in this study is openly available online.
